# Male-specific association between *MT-ND4* 11719 A/G polymorphism and ulcerative colitis: a mitochondria-wide genetic association study

**DOI:** 10.1186/s12876-016-0509-1

**Published:** 2016-10-03

**Authors:** Theresa Dankowski, Torsten Schröder, Steffen Möller, Xinhua Yu, David Ellinghaus, Florian Bär, Klaus Fellermann, Hendrik Lehnert, Stefan Schreiber, Andre Franke, Christian Sina, Saleh M. Ibrahim, Inke R. König

**Affiliations:** 1 Institute of Medical Biometry and Statistics, University of Lübeck, University Hospital Schleswig-Holstein, Campus Lübeck, Ratzeburger Allee 160, 23562 Lübeck, Germany; 2 Department of Medicine I, University of Lübeck, Ratzeburger Allee 160, 23538 Lübeck, Germany; 3 Institute for Systemic Inflammation Research, University of Lübeck, Ratzeburger Allee 160, 23538 Lübeck, Germany; 4 The Lübeck Institute of Experimental Dermatology, University of Lübeck, Ratzeburger Allee 160, 23538 Lübeck, Germany; 5 Institute for Biostatistics and Informatics in Medicine and Ageing Research, Rostock University Medical Center, Ernst-Heydemann-Straße 8, 18057 Rostock, Germany; 6 Priority Area Asthma & Allergy, Research Center Borstel, Airway Research Center North (ARCN), Members of the German Center for Lung Research (DZL), Parkallee 1-40, 23845 Borstel, Germany; 7 Xiamen-Borstel Joint Laboratory of Autoimmunity, Medical College of Xiamen University, Xiamen, 361102 China; 8 Institute of Clinical Molecular Biology, Christian-Albrechts-University of Kiel, Schittenhelmstraße 12, 24105 Kiel, Germany

**Keywords:** Mitochondrial gene polymorphism, Ulcerative colitis, Male-specific association, Mucosal energy deficiency, Mitochondria-wide association study, *MT-ND4*, rs2853495, ATP, Haplogroup HV

## Abstract

**Background:**

Ulcerative colitis (UC) is a chronic inflammatory disorder of still unknown pathogenesis. Increasing evidence indicates that alterations in mitochondrial respiration and thus adenosine triphosphate (ATP) production are involved. This may contribute to mucosal energy deficiency and subsequently intestinal barrier malfunction, which is accepted to be a major hallmark of UC. Genetic alterations of the mitochondrial genome are one cause of mitochondrial dysfunction. However, less is known about mitochondrial gene polymorphisms in UC. Therefore, we aimed at identifying genetic associations between mitochondrial polymorphisms and UC.

**Methods:**

German UC cases (*n* = 1062) and German healthy controls (*n* = 3030) were genotyped using the Affymetrix Genome-Wide Human SNP Array 6.0. The primary association analysis was to test for associations between mitochondrial single nucleotide polymorphisms (SNPs) and UC using Fisher’s exact test in the total sample and stratified by sex. In addition, we tested for associations between mitochondrial haplogroups and UC and for interactions between the most promising mitochondrial SNPs and nuclear SNPs. An independent set of German subjects with 1625 UC cases and 3575 controls was used for replication.

**Results:**

We identified a genetic association between the *MT-ND4* 11719 A/G polymorphism and UC in the subgroup of males (rs2853495; odds ratio, 1.40; 95 % confidence interval, 1.13 to 1.73; *p* = 0.002). This association was replicated in the second independent cohort. In the association analysis based on mitochondrial haplogroups the lowest *p* values were reached for haplogroups HV and T (*p* = 0.029 and 0.035). Haplogroup HV is determined by the mitochondrial 11719 A/G polymorphism. Accordingly, this association was only found in the subgroup of males (*p* = 0.009).

**Conclusions:**

For the first time, we observed an association between the *MT-ND4* 11719 A/G polymorphism and UC. The gene *MT-ND4* encodes for a subunit of the mitochondrial electron transport chain complex I, which is pivotal for ATP production and might therefore contribute to mucosal energy deficiency. The male-specific association indicates differences between males and females concerning the impact of mitochondrial gene polymorphisms on the development of UC. Further investigations of the functional mechanism underlying this association and the relevance of the gender-specificity are highly warranted.

**Electronic supplementary material:**

The online version of this article (doi:10.1186/s12876-016-0509-1) contains supplementary material, which is available to authorized users.

## Background

Ulcerative colitis (UC) belongs to the family of inflammatory bowel diseases (IBD). In western countries, UC exhibits rising prevalence with a slight predominance for males [1]. UC is identified by chronic and recurrent intestinal inflammation of yet unknown origin. The present pathophysiological concept suggests a disturbed intestinal barrier due to a combination of factors, including dysregulation of the host’s mucosal immune system, environmental factors, changes in the intestinal microbiome and a genetic susceptibility [2–4]. Furthermore, mucosal adenosine triphosphate (ATP) levels are reduced in patients with UC [5, 6]. Since mitochondria are the primary source of cellular ATP, this implicates a pathophysiological relevance of mitochondrial dysfunction.

Mitochondrial dysfunction is commonly caused by single nucleotide polymorphism (SNPs) of the mitochondrial genome and inadequate repair mechanisms [7]. Monogenic mutations of mitochondrial genes are known to cause severe mitochondrial dysfunction leading to rare and multisystemic diseases like Kearns Sayre Syndrome, Leigh-syndrome or Leber’s Hereditary Optic Neuropathy [8–11]. According to the current knowledge, these syndromes are not associated with UC. However, the mitochondrial DNA codes for mitochondrial oxidative phosphorylation proteins and consequently may be of high relevance for the cellular energy supply. Thus, cells with high need of energy to persevere organ function are highly susceptible to mutations of the mitochondrial genome. Many cell types of the intestinal tract, such as epithelial cells, muscle cells, and immune cell, have high-energy requirements. Accordingly, it is highly likely that the mitochondrial DNA and respective variants may be involved in gastrointestinal symptoms. In fact, gastrointestinal symptoms of irritable bowel syndrome could already been linked to mitochondrial polymorphisms [12]. Furthermore, there is increasing evidence for a role of mitochondrial gene polymorphisms in the development of common diseases such as type 2 diabetes mellitus, neurodegenerative diseases and several different types of cancer [13–16].

We recently described that variants in mitochondrial DNA determine mucosal ATP levels and susceptibility to experimental colitis in mice [17]. In addition, in a small European UC cohort a mitochondrial polymorphism was just recently described to be associated with UC [18]. However, mitochondrial SNPs are usually excluded from genetic associations studies, so we aimed at investigating the role of mitochondrial gene variations in the development of UC. Here, we report on a mitochondria-wide genetic association study using a large German UC study group and population-based controls. The restriction to German subjects has the advantage that the German population is known to be genetically quite homogenous [19]. As a result, a male-specific association between a genetic variation of the *MT-ND4* gene (11719 A/G, rs2853495) and UC could be identified and replicated in another independent set of German subjects.

## Methods

### Study subjects

In total, 2687 German UC patients were available for this study who were recruited at the Department of General Internal Medicine of the Christian-Albrechts-University Kiel, the Charité University Hospital Berlin, through local outpatient services, and nationwide with support of the German Crohn and Colitis Foundation. Of these, 1043 German patients were previously used in a genome-wide association study (GWAS) for UC [20]. Clinical, radiological, histological, and endoscopic (i.e. type and distribution of lesions) examinations were required to unequivocally confirm the diagnosis of UC [21]. Data from 1062 out of all 2687 UC cases composed of 459 males and 603 females were used in the initial study (more characteristics in Additional file 1: Table S1). Data of the remaining 1625 UC cases composed of 706 males, 914 females and 5 patients with missing sex were used for replication.

For the initial study 1795 healthy controls were selected from the KORA F4 survey [22], which is an independent population-based sample of the general population living in the region of Augsburg, Southern Germany. In addition, data from 1240 German control individuals was obtained from the Popgen biobank [23] for the initial study resulting in a total of 3035 controls (1565 males, 1470 females). For the replication study, data from 3575 controls from Popgen biobank composed of 1701 males and 1874 females were used. Of note, 1214 controls subjects from the Popgen biobank and 489 controls from the KORA survey, respectively, had been part of the previous GWAS [20].

### Genotyping

Single nucleotide polymorphism (SNP) genotyping in the discovery panel was performed using the Affymetrix® Genome-Wide Human SNP Array 6.0 (for details see [20]). Genotype calling was performed using the Birdseed v2 algorithm implemented in Affymetrix Power Tools version 1.12.0. Prior to genotype calling, samples with a low contrast-quality control value (contrast-qc < 0.40) were excluded.

### Quality control

For quality control on individual level in the initial study group we excluded samples with a call fraction < 90 % for the mitochondrial SNPs. This applied to five control individuals resulting in data for 1062 UC cases and 3030 controls. On mitochondrial marker level we excluded SNPs with MAF < 0.5 %. This applied to 60 of the initial 119 SNPs. Nine SNPs were excluded because of a call fraction < 90 % separate for cases and controls and two SNPs because they were wrongly assigned to the mitochondrial DNA. Furthermore, we investigated the cluster plots of the remaining mitochondrial SNPs and excluded 21 SNPs with critical issues. In conclusion, 27 mitochondrial SNPs passed our quality control. In Europeans 144 variants with a frequency >1 % were identified [13]. 37 out of these 144 variants are captured or partly captured by our 27 SNPs after quality control.

For quality control on nuclear marker level we excluded SNPs with MAF < 1 %, call fraction < 98 % separate for cases and controls and *p* value < 10^−04^ for test on Hardy Weinberg equilibrium in the controls. We also excluded SNPs on the X or Y chromosome. 546,808 of the initial 934,968 nuclear SNPs passed our quality control.

### Statistical analysis

Our primary association analysis in the initial study group was based on testing for associations between single SNPs and the UC phenotype by applying Fisher’s exact test. This was done for the total initial study group and separate for males and females. The most promising SNPs from this approach with *p* value < 0.01 were taken forward to replication. To analyze joint effects across both stages, the Cochran Mantel Haenszel test was used. The global significance level is set to 0.05, which corresponds to a local significance level of 0.05/(27⋅3) = 6.2⋅10^−04^ for testing 27 SNPs in three groups.

For secondary association analysis we reconstructed haplogroups. The haplogroup assignment was conducted by using the web application HaploGrep [24] that is based on Phylotree [25]. According to the recommendations we only included haplogroup assignments with a quality score above 90 % as determined by HaploGrep. The only exception was haplogroup H2a2a, whose quality score was 0 %, because this is the haplogroup of the reference sequence rCRS. This yielded haplogroup assignments with good quality for 1059 cases and 3007 controls. Although HaploGrep gives very specific subhaplogroups, we only used the corresponding main haplogroups that are most common in Europe (H, I, J, K, T, U, V, W, X, HV, JT) for further analysis. We compared the relative frequencies of the most common European haplogroups in our sample to the frequencies given by Mitomap [26] and found good agreement (Additional file 2: Table S2). We only included European haplogroups with a frequency above 2 % and used Fisher’s exact test to test each haplogroup against all other haplogroups in the total initial study group and separate for males and females. Haplogroups H, V and HV were examined as one group named HV because of their close relationship. Allele counts for all markers in the tested haplogroups are provided in Additional file 3: Table S3.

The functional relevance of the mitochondrial SNP rs2853495 may be affected by the nuclear genome. Thus, to further explore our findings, we tested for an interaction between the most promising mitochondrial SNP rs2853495 and nuclear SNPs in the total initial study group and the subgroup of males. We used a logistic regression model for each nuclear SNP that included the main effects of the nuclear and the mitochondrial SNPs and the interaction term. We modeled additive effects for the nuclear SNPs and we used a 0, 1 allele dosage coding for the mitochondrial SNPs. A genome-wide significance level of 5⋅10^−08^ was used for the interaction analysis.

### Software

We used the free software R [27] and Plink [28] for quality control and analysis.

## Results

Table 1 lists SNPs with a *p* value < 0.01 in the total initial sample or in one of the sex-specific subgroups. Association plots for all SNPs in the total initial sample and both sex-specific subgroups are shown in Additional file 4: Figure S1, Additional file 5: Figure S2 and Additional file 6: Figure S3. The association of the SNP rs2853495 in the gene *MT-ND4* with UC was successfully validated in the total sample, and the combined analyses of this SNP across both stages yielded a *p* value of 1.9⋅10^−4^ (odds ratio (OR), 1.19; 95 % confidence interval (95 % CI), 1.09 to 1.30). This is significant on a global significance level of 0.05, which corresponds to a local significance level of 0.05/(27⋅3) = 6.2⋅10^−4^ adjusted for testing of 27 mitochondrial SNPs in three groups. In the sex-specific analyses the association between rs2853495 and UC was only identified in the subgroup of males. This was also validated using the male subgroup of the replication data. The combined analyses across both stages yielded a significant *p* value of 1.5⋅10^−5^ (OR, 1.35; 95 % CI, 1.18 to 1.55). Notably, the *p* value was smaller in the subgroup of males even though the sample size was smaller than in the total initial sample.

**Table 1:** Results from the single SNP association analysis for SNPs with a two-sided *p* value < 0.01 from Fisher’s exact test in the initial sample or in one of the sex-specific subgroups

mtSNP^a^	BP^b^	A1^c^	A2^d^	Subsample	Initial analysis				Replication				Combined^g^	
					p	OR^e^	A1^c^ frequency		p	OR^e^	A1 frequency		p	OR^e^
							Cases	Ctrls^f^			Cases	Ctrls^f^		
rs2853495	11719	G	A	Total	0.007	1.22 [1.06; 1.40]	0.528	0.478	0.011	1.17 [1.04; 1.31]	0.525	0.486	1.921⋅10^−4^	1.19 [1.09; 1.30]
				Males	0.002	1.40 [1.13; 1.73]	0.562	0.479	0.002	1.32 [1.10; 1.57]	0.551	0.482	1.512⋅10^−5^	1.35 [1.18; 1.55]
				Females	0.328	1.10 [0.91; 1.34]	0.502	0.477	–	–	–	–	–	–
rs3899498	13368	A	G	Total	0.005	0.71 [0.56; 0.91]	0.085	0.115	0.334	0.90 [0.74; 1.10]	0.091	0.100	1.012⋅10^−2^	0.82 [0.70; 0.95]
				Males	0.126	0.75 [0.53; 1.07]	0.090	0.117	–	–	–	–	–	–
				Females	0.026	0.68 [0.49; 0.96]	0.080	0.113	–	–	–	–	–	–

^a^Mitochondrial single nucleotide polymorphism; ^b^Base-pair position according to UCSC version hg19; ^c^Effect allele; ^d^Second allele; ^e^Odds ratio with 95 % confidence interval; ^f^Controls; ^g^Combined analysis of initial and replication data

The second approach was to test for association between mitochondrial haplogroups and UC (see Table 2). The lowest *p* values in the total sample were reached for haplogroups HV and T (*p* = 0.029 and 0.035), in which haplogroup HV is determined by the mitochondrial 11719 A/G polymorphism. Accordingly, the association to haplogroup HV was only found in the subgroup of males (*p* = 0.009).

**Table 2:** Results of the haplogroup based analysis with frequencies of cases and controls in the total sample and *p* values from Fisher’s exact test

Haplogroup	Control	Cases	p^a^	p_males_^b^	p_females_^c^
HV	48.05	52.03	0.029	0.009	0.498
U	15.96	15.30	0.624	0.056	0.322
J	9.94	9.82	0.952	0.060	0.147
T	11.64	9.25	0.035	0.357	0.060
K	7.02	7.08	0.944	1.000	0.929

^a^ *p* value in the total sample, ^b^ *p* value in the subgroup of males, ^c^ *p* value in the subgroup of females

No interaction between SNP rs2853495 and a nuclear SNP reached genome-wide significance, i.e. *p* < 5⋅10^−8^, in the total initial sample but some of the results merit further attention. Specifically, a cluster of SNPs on chromosome 3 shows interaction with rs2853495 (see Table 3). The results for the top 50 nuclear SNPs according to the *p* value for interaction are shown in Additional file 7: Table S4. Additional file 8: Table S5 shows the results for all nuclear SNPs with interaction *p* value < 1⋅10^−04^ in the subgroup of males.

**Table 3:** Results for the top 10 nuclear SNPs on chromosome 3 according to *p* value for interaction with rs2853495, sorted by chromosomal positions

Chr^a^	Nuclear SNP	BP^b^	A1^c^	p			Gene
				Nuclear^d^	Mito^e^	Interaction^f^	
3	rs7620175	35155767	T	6.58⋅10^−02^	4.25⋅10^−01^	8.47⋅10^−06^	*LOC101928135*
	rs12493494	60412390	T	1.41⋅10^−03^	4.37⋅10^−06^	1.57⋅10^−05^	*FHIT*
	rs505014	62731917	G	2.12⋅10^−04^	1.11⋅10^−01^	5.78⋅10^−05^	*CADPS*
	rs498746	62731985	T	2.64⋅10^−04^	1.48⋅10^−01^	9.26⋅10^−05^	*CADPS*
	rs4676732	121314320	T	2.74⋅10^−02^	9.31⋅10^−02^	6.99⋅10^−05^	*FBXO40*
	rs6784995	122938098	A	7.04⋅10^−04^	9.13⋅10^−07^	3.37⋅10^−05^	*SEC22A*
	rs9289215	122962809	A	2.72⋅10^−03^	1.37⋅10^−06^	8.36⋅10^−05^	*SEC22A*
	rs6784930	123001494	A	1.76⋅10^−02^	1.21⋅10^−06^	1.03⋅10^−05^	*ADCY5*
	rs17809756	159630084	A	4.30⋅10^−06^	7.33⋅10^−01^	7.79⋅10^−06^	Near *SCHIP1*, *ILI2A*
	rs6800685	190139275	T	5.61⋅10^−02^	2.10⋅10^−01^	1.12⋅10^−05^	Near *CLDN16*, *TMEM207*

^a^Chromosome; ^b^Base-pair position according to UCSC version hg19; ^c^Minor allele; ^d^Main effect of nuclear SNP; ^e^Main effect of mitochondrial SNP; ^f^Interaction term

## Discussion

As the salient finding of this study, we identified an association between a homoplastic mitochondrial DNA variation in the gene *MT-ND4* (11719 A/G, rs2853495) with male UC patients. This association was even validated in a second independent sample, and the combined value is significant after controlling for the multiple testing of 27 mitochondrial SNPs in three groups.

The here reported mtDNA variation (11719 A/G, rs2853495) affects the gene *MT-ND4*, which encodes for one subunit of the NADH dehydrogenase 4 of complex I of the mitochondrial respiratory chain. Since this endosymbiosis of mitochondria eukaryotic cells, the mitochondria have lost most of their genetic material to the cell nucleus. However, mitochondria still retained some genes in their own genome, which are highly essential for cellular respiration and ATP generation [29]. The gene *MT-ND4* encodes for one subunit of the NADH dehydrogenase 4 as a part of the mitochondrial oxidative phosphorylation system (OXPHOS) complex I. Complex I consists of 44 different subunits, seven of which are mitochondrially encoded [30]. The function of complex I as first step of the electron transport chain is to extract electrons from NADH and donate them to ubiquinone. This reaction releases energy, which is used to transport protons across the mitochondrial inner membrane. In this way, complex I contributes to the maintenance of the proton gradient, which fuels mitochondrial ATP production and many other mitochondrial functions [31]. Mutations in the *MT-ND4* gene may have an important influence on mitochondrial respiratory chain function and therefore a genetic variation of this gene may lead to alterations of the cellular energy metabolism. Consequently, a mutation in this mitochondrial gene may contribute among many other factors to mitochondrial dysfunction and mucosal energy deficiency, which has been detected in UC patients [5, 6].

The here identified DNA variation in *MT-ND4* has been previously shown to be associated with numerous classical mitochondrial disorders like Leber’s Hereditary Optic Neuropathy [32] or Leigh syndrome [30]. Moreover, dysfunction of *MT-ND4* has been described in the context of experimental autoimmune encephalomyelitis, which is an animal model of multiple sclerosis [33], breast cancer [34], cystic fibrosis [35] and other diseases. Most interestingly, a recent report in a small cohort of UC patients could bring a different mitochondrial DNA variant affecting *MT-ND4* into relation to UC, which underlines the possible relevance for intestinal homeostasis [18]. Of note, we found the association only for male subjects. In fact, there are several studies reporting UC to be more frequent in males [1, 36, 37]. Moreover, it was observed that males responded less to a three months corticosteroidal therapy in terms of mucosal healing [38]. Beside these reports, it is well known that male mice respond aggravated to experimental colitis [39]. However, it remains speculation whether mitochondrial gene polymorphisms may be implicated in these clinical observations. Therefore, we alternatively suggest the presence of cofactors functionally cooperating with the polymorphism of *MT-ND4*, which increase the susceptibility for UC. These cofactors could for instance be nuclear encoded genes. Accordingly, we studied interactions between the identified mitochondrial SNP and nuclear SNPs. As apparent in Additional file 7: Table S4 and Additional file 8: Table S5 no genome-wide significant interaction was found. Nevertheless, interesting candidates emerged from these analyses. These SNPs merit further investigation due to their involvement in highly energy-dependent pathways such as Calcium-dependent secretion activator 1 (*CADPS*) and the Vesicle-trafficking protein *SEC22A* [40, 41]. However, more detailed data, e.g. provided by deep sequencing, are necessary to answer the question, whether there are functionally active interactions between mitochondrial and nuclear gene variations.

Considering that homoplasmic mitochondrial DNA variants, like the here identified polymorphism, frequently determine haplogroups [42], we tested for associations between different mitochondrial haplogroups and UC. The lowest *p* values were reached for the mitochondrial haplogroups HV and T (*p* = 0.029 and 0.035). Notably, the mitochondrial 11719 A/G polymorphism signifies the haplogroup HV [43]. Considering that these haplogroups occur approximately in 40-50 % of modern Eurasian ethnicity, in which the incidence of IBD is particularly high [44, 45], these results might further point to a critical role of the mitochondrial genome for the pathogenesis of UC. Haplogroup T is additionally defined by a signature SNP of complex I [46, 47]. Haplogroup T is currently found with high concentration in the eastern Baltic Sea region, in which the incidence of IBD is emerging [45]. Whether this might be a functional connection of mitochondrial haplogroups and UC or just a coincidence must be investigated in the future.

## Conclusions

We identified and replicated a yet unknown male-specific association between a mitochondrial gene polymorphism in *MT-ND4* (11719 A/G, rs2853495) and UC. This indicates that mitochondrial genetics may determine gender-specific differences in disease prevalence and therapeutical response. Consequently, this study may help to deepen the knowledge of UC pathology and clinical course. Further studies are required to recapitulate the association of mitochondrial gene polymorphisms and UC and to elucidate the definite role of the mitochondrial genome in UC development.

## Abbreviations

ADP, adenosine diphosphate; ATP, adenosine triphosphate; GWAS, genome-wide association study; IBD, inflammatory bowel diseases; MAF, minor allele frequency; OXPHOS, oxidative phosphorylation; SNP, single-nucleotide polymorphism; UC, ulcerative colitis

## Additional files

Additional file 1: Table S1. Characteristics of UC cases used in the initial analysis. (DOC 42 kb) Additional file 2: Table S2. Haplogroup frequencies in our sample and given by Mitomap (http://www.mitomap.org/bin/view.pl/MITOMAP/HaplogroupMarkers, last accessed 15/09/2014). (DOC 43 kb) Additional file 3: Table S3. Allele counts in the tested haplogroups assigned with HaploGrep (Kloss-Brandstätter 2011 Hum Mutat 32:25–32). Base pair position and allele are shown for all markers. (DOC 107 kb) Additional file 4: Figure S1. Results of SNP based association analysis in the total initial sample. Horizontal line located at 0.01. (DOC 33 kb) Additional file 5: Figure S2. Results of SNP based association analysis in the male subgroup of the initial sample. Horizontal line located at 0.01. (DOC 34 kb) Additional file 6: Figure S3. Results of SNP based association analysis in the female subgroup of the initial sample. Horizontal line located at 0.01. (DOC 33 kb) Additional file 7: Table S4. Results for the top 50 nuclear SNPs according to *p* value for interaction with rs2853495 in the total initial sample, sorted by chromosomal positions. (DOC 86 kb) Additional file 8: Table S5. Results for nuclear SNPs with *p* value < 1·10^−04^ for interaction with rs2853495 in the subgroup of males, sorted by chromosomal positions. (DOC 78 kb) Additional file 9: Data in aggregate form. (XLSX 16 kb)
