# Supplementary material for: Male-specific association between MT-ND4 11719 A/G polymorphism and ulcerative colitis: a mitochondria-wide genetic association study
Source: BMC Gastroenterol. 2016 Oct 3;16:118. doi: 10.1186/s12876-016-0509-1 (PMC5048482; doi:10.1186/s12876-016-0509-1)
Supplement: Additional file 1: Table S1. — Characteristics of UC cases used in the initial analysis. (DOC 42 kb) [file 12876_2016_509_MOESM1_ESM.doc]

**Table S1:** Characteristics of UC cases used in the initial analysis.

| **Sex distribution** | | | |
| --- | --- | --- | --- |
|  | females, n | males, n | missing, n |
|  | 603 | 459 | 0 |
| **Age distribution** (years) | | | |
|  | mean | standard deviation | missing, n |
| at sampling | 43.2 | 14.2 | 45 |
| at onset | 27.1 | 12.7 | 186 |
| **Disease extent** | | | |
|  | yes, n | no, n | missing, n |
| left sided colitis | 601 | 252 | 209 |
| extensive colitis | 196 | 356 | 510 |
| colectomy | 23 | 422 | 617 |
| **Smoking habit** | | | |
|  | current or previous, n | never, n | missing, n |
|  | 398 | 481 | 183 |
