# Supplementary material for: Male-specific association between MT-ND4 11719 A/G polymorphism and ulcerative colitis: a mitochondria-wide genetic association study
Source: BMC Gastroenterol. 2016 Oct 3;16:118. doi: 10.1186/s12876-016-0509-1 (PMC5048482; doi:10.1186/s12876-016-0509-1)
Supplement: Additional file 2: Table S2. — Haplogroup frequencies in our sample and given by Mitomap (http://www.mitomap.org/bin/view.pl/MITOMAP/HaplogroupMarkers, last accessed 15/09/2014). (DOC 43 kb) [file 12876_2016_509_MOESM2_ESM.doc]

**Table S2:** Haplogroup frequencies in our sample and given by Mitomap (<http://www.mitomap.org/bin/view.pl/MITOMAP/HaplogroupMarkers>, last accessed 15/09/2014).

| Haplogroup | Mitomap | Our sample | | |
| --- | --- | --- | --- | --- |
|  |  | Controls | Cases | Total |
| H | 41 | 42.20 | 46.18 | 43.24 |
| U | 18 | 15.96 | 15.30 | 15.79 |
| J | 9 | 9.94 | 9.82 | 9.91 |
| T | 8 | 11.64 | 9.25 | 11.02 |
| V | 7 | 2.89 | 3.68 | 3.10 |
| K | 5 | 7.02 | 7.08 | 7.03 |
| X | 2 | 0.07 |  | 0.05 |
| I | 2 |  |  |  |
| W | 2 |  |  |  |
| JT |  | 0.03 |  | 0.02 |
| HV |  | 2.96 | 2.17 | 2.75 |
| Other | 7 | 7.28 | 6.52 | 7.08 |
