# Supplementary material for: Male-specific association between MT-ND4 11719 A/G polymorphism and ulcerative colitis: a mitochondria-wide genetic association study
Source: BMC Gastroenterol. 2016 Oct 3;16:118. doi: 10.1186/s12876-016-0509-1 (PMC5048482; doi:10.1186/s12876-016-0509-1)
Supplement: Additional file 3: Table S3. — Allele counts in the tested haplogroups assigned with HaploGrep (Kloss-Brandstätter 2011 Hum Mutat 32:25–32). Base pair position and allele are shown for all markers. (DOC 107 kb) [file 12876_2016_509_MOESM3_ESM.doc]

**Table S3:** Allele counts in the tested haplogroups assigned with HaploGrep (Kloss-Brandstätter 2011 Hum Mutat 32:25-32). Base pair position and allele are shown for all markers.

| **Marker** | **Haplogroup** | | | | |
| --- | --- | --- | --- | --- | --- |
|  | HV | U | J | T | K |
| 1438A | 130 | 1 | 0 | 2 | 0 |
| 1438G | 1865 | 641 | 403 | 446 | 286 |
| 1598A | 5 | 0 | 0 | 14 | 0 |
| 1598G | 1991 | 642 | 403 | 434 | 286 |
| 1811A | 1985 | 393 | 399 | 445 | 13 |
| 1811G | 11 | 249 | 4 | 3 | 273 |
| 2706A | 1756 | 14 | 12 | 6 | 4 |
| 2706G | 240 | 628 | 391 | 442 | 282 |
| 3010A | 668 | 22 | 307 | 0 | 0 |
| 3010G | 1328 | 619 | 96 | 448 | 286 |
| 3197C | 5 | 370 | 0 | 0 | 0 |
| 3197T | 1989 | 272 | 403 | 448 | 286 |
| 4580A | 126 | 0 | 1 | 0 | 0 |
| 4580G | 1869 | 642 | 402 | 448 | 286 |
| 4769A | 122 | 0 | 0 | 0 | 0 |
| 4769G | 1872 | 642 | 403 | 448 | 286 |
| 9698C | 0 | 18 | 0 | 0 | 286 |
| 9698T | 1995 | 624 | 403 | 448 | 0 |
| 10238T | 1996 | 642 | 403 | 446 | 274 |
| 10463C | 8 | 0 | 4 | 448 | 0 |
| 10463T | 1988 | 642 | 399 | 0 | 286 |
| 10550A | 1996 | 642 | 403 | 448 | 0 |
| 10550G | 0 | 0 | 0 | 0 | 286 |
| 10589A | 41 | 1 | 1 | 0 | 1 |
| 10589G | 1954 | 641 | 400 | 448 | 285 |
| 11467A | 1995 | 0 | 403 | 448 | 1 |
| 11467G | 0 | 638 | 0 | 0 | 283 |
| 11719A | 24 | 628 | 396 | 443 | 281 |
| 11719G | 1963 | 2 | 0 | 0 | 0 |
| 11914A | 20 | 1 | 0 | 13 | 25 |
| 11914G | 1973 | 632 | 402 | 434 | 259 |
| 12007A | 3 | 0 | 34 | 6 | 0 |
| 12007G | 1993 | 642 | 368 | 442 | 286 |
| 13368A | 0 | 0 | 0 | 434 | 0 |
| 13368G | 1994 | 642 | 403 | 2 | 286 |
| 13617C | 0 | 365 | 0 | 0 | 0 |
| 13617T | 1996 | 275 | 403 | 448 | 286 |
| 13708A | 25 | 4 | 399 | 1 | 2 |
| 13708G | 1969 | 638 | 0 | 447 | 283 |
| 14905A | 3 | 0 | 0 | 447 | 0 |
| 14905G | 1993 | 642 | 403 | 0 | 286 |
| 15043A | 1 | 1 | 0 | 31 | 0 |
| 15043G | 1994 | 641 | 403 | 417 | 286 |
| 15301A | 4 | 3 | 0 | 0 | 5 |
| 15301G | 1992 | 639 | 403 | 448 | 281 |
| 15326A | 46 | 3 | 0 | 1 | 0 |
| 15326G | 1950 | 639 | 403 | 447 | 286 |
| 15452A | 0 | 0 | 401 | 440 | 0 |
| 15452C | 1994 | 638 | 0 | 5 | 286 |
| 15607A | 1994 | 641 | 403 | 1 | 286 |
| 15607G | 0 | 1 | 0 | 447 | 0 |
| 15784C | 14 | 6 | 0 | 0 | 0 |
| 15784T | 1982 | 636 | 403 | 447 | 286 |
