# Supplementary material for: Male-specific association between MT-ND4 11719 A/G polymorphism and ulcerative colitis: a mitochondria-wide genetic association study
Source: BMC Gastroenterol. 2016 Oct 3;16:118. doi: 10.1186/s12876-016-0509-1 (PMC5048482; doi:10.1186/s12876-016-0509-1)
Supplement: Additional file 6: Figure S3. — Results of SNP based association analysis in the female subgroup of the initial sample. Horizontal line located at 0.01. (DOC 33 kb) [file 12876_2016_509_MOESM6_ESM.doc]

**Figure S3:** Results of SNP based association analysis in the female subgroup of the initial sample. Horizontal line located at 0.01.
