# Supplementary material for: Male-specific association between MT-ND4 11719 A/G polymorphism and ulcerative colitis: a mitochondria-wide genetic association study
Source: BMC Gastroenterol. 2016 Oct 3;16:118. doi: 10.1186/s12876-016-0509-1 (PMC5048482; doi:10.1186/s12876-016-0509-1)
Supplement: Additional file 7: Table S4. — Results for the top 50 nuclear SNPs according to p value for interaction with rs2853495 in the total initial sample, sorted by chromosomal positions. (DOC 86 kb) [file 12876_2016_509_MOESM7_ESM.doc]

**Table S4:** Results for the top 50 nuclear SNPs according to p value for interaction with rs2853495 in the total initial sample, sorted by chromosomal positions.

| Chr^a^ | Nuclear SNP | Position^b^ | A1^c^ | p value | | | Gene |
| --- | --- | --- | --- | --- | --- | --- | --- |
|  |  |  |  | nuclear^d^ | mito^e^ | interaction^f^ |  |
| 1 | rs9729667 | 78349214 | T | 2.49⋅10^-02^ | 1.47⋅10^-06^ | 5.42⋅10^-05^ | *NEXN-AS1* |
| 2 | rs12691853 | 134692470 | A | 7.36⋅10^-03^ | 7.61⋅10^-01^ | 6.70⋅10^-05^ | *Near NCKAP5* |
|  | rs13388210 | 134699298 | C | 1.54⋅10^-02^ | 5.26⋅10^-01^ | 4.42⋅10^-05^ |  |
|  | rs4299382 | 134705923 | C | 3.81⋅10^-02^ | 5.28⋅10^-01^ | 3.69⋅10^-05^ |  |
|  | rs1026983 | 134706575 | G | 3.66⋅10^-02^ | 7.66⋅10^-01^ | 6.56⋅10^-05^ |  |
|  | rs2087006 | 134713735 | G | 2.85⋅10^-02^ | 4.96⋅10^-01^ | 2.05⋅10^-05^ |  |
|  | rs10184572 | 226255655 | T | 2.72⋅10^-03^ | 2.95⋅10^-06^ | 2.90⋅10^-05^ |  |
|  | rs6753020 | 226299309 | C | 7.71⋅10^-03^ | 4.66⋅10^-06^ | 3.28⋅10^-05^ | *NYAP2* |
| 3 | rs7620175 | 35155767 | T | 6.58⋅10^-02^ | 4.25⋅10^-01^ | 8.47⋅10^-06^ |  |
|  | rs12493494 | 60412390 | T | 1.41⋅10^-03^ | 4.37⋅10^-06^ | 1.57⋅10^-05^ |  |
|  | rs505014 | 62731917 | G | 2.12⋅10^-04^ | 1.11⋅10^-01^ | 5.78⋅10^-05^ | *CADPS* |
|  | rs498746 | 62731985 | T | 2.64⋅10^-04^ | 1.48⋅10^-01^ | 9.26⋅10^-05^ | *CADPS* |
|  | rs4676732 | 121314320 | T | 2.74⋅10^-02^ | 9.31⋅10^-02^ | 6.99⋅10^-05^ | *FBXO40* |
|  | rs6784995 | 122938098 | A | 7.04⋅10^-04^ | 9.13⋅10^-07^ | 3.37⋅10^-05^ | *SEC22A* |
|  | rs9289215 | 122962809 | A | 2.72⋅10^-03^ | 1.37⋅10^-06^ | 8.36⋅10^-05^ | *SEC22A* |
|  | rs6784930 | 123001494 | A | 1.76⋅10^-02^ | 1.21⋅10^-06^ | 1.03⋅10^-05^ |  |
|  | rs17809756 | 159630084 | A | 4.30⋅10^-06^ | 7.33⋅10^-01^ | 7.79⋅10^-06^ | *Near SCHIP1, ILI2A* |
|  | rs6800685 | 190139275 | T | 5.61⋅10^-02^ | 2.10⋅10^-01^ | 1.12⋅10^-05^ |  |
| 4 | rs7664766 | 36013509 | A | 8.24⋅10^-03^ | 1.33⋅10^-04^ | 7.47⋅10^-05^ |  |
|  | rs10049599 | 36017518 | G | 1.07⋅10^-02^ | 1.15⋅10^-04^ | 4.54⋅10^-05^ |  |
|  | rs1776515 | 37188229 | C | 1.72⋅10^-03^ | 8.00⋅10^-01^ | 5.44⋅10^-05^ |  |
|  | rs1656207 | 37211990 | T | 1.60⋅10^-03^ | 8.17⋅10^-01^ | 6.44⋅10^-05^ |  |
| 5 | rs10940060 | 65712525 | A | 1.04⋅10^-05^ | 2.90⋅10^-01^ | 8.82⋅10^-05^ |  |
|  | rs10514994 | 65739556 | A | 3.77⋅10^-05^ | 2.87⋅10^-01^ | 4.66⋅10^-05^ |  |
| 7 | rs2644311 | 2845371 | A | 3.18⋅10^-06^ | 1.95⋅10^-01^ | 2.27⋅10^-05^ |  |
|  | rs2163640 | 7168493 | G | 2.28⋅10^-02^ | 1.94⋅10^-05^ | 3.96⋅10^-05^ |  |
|  | rs2057986 | 22098381 | G | 1.50⋅10^-03^ | 1.60⋅10^-05^ | 9.33⋅10^-05^ |  |
|  | rs2367910 | 81786043 | A | 1.12⋅10^-02^ | 1.18⋅10^-05^ | 1.33⋅10^-05^ |  |
| 8 | rs16903111 | 129586776 | C | 3.65⋅10^-04^ | 1.88⋅10^-05^ | 5.81⋅10^-05^ |  |
| 9 | rs4742307 | 7093689 | G | 2.24⋅10^-01^ | 8.91⋅10^-01^ | 5.34⋅10^-05^ | *KDM4C* |
|  | rs10976048 | 7121029 | G | 1.40⋅10^-01^ | 7.38⋅10^-01^ | 1.87⋅10^-05^ | *KDM4C* |
|  | rs10976053 | 7126175 | G | 2.38⋅10^-01^ | 7.94⋅10^-01^ | 2.97⋅10^-05^ | *KDM4C* |
|  | rs10815527 | 7132610 | C | 1.80⋅10^-01^ | 5.50⋅10^-01^ | 1.32⋅10^-05^ | *KDM4C* |
|  | rs4842074 | 138913404 | A | 6.74⋅10^-03^ | 5.04⋅10^-05^ | 7.37⋅10^-05^ |  |
| 11 | rs2726413 | 99300137 | A | 2.31⋅10^-02^ | 1.32⋅10^-01^ | 6.75⋅10^-05^ |  |
|  | rs11601707 | 134619154 | C | 1.09⋅10^-02^ | 2.35⋅10^-06^ | 5.34⋅10^-05^ |  |
| 12 | rs959926 | 107071637 | A | 1.47⋅10^-02^ | 4.30⋅10^-01^ | 6.07⋅10^-05^ | *RFX4* |
|  | rs10507212 | 107071911 | A | 2.18⋅10^-02^ | 4.40⋅10^-01^ | 7.11⋅10^-05^ | *RFX4* |
|  | rs7300105 | 107141967 | G | 3.65⋅10^-02^ | 4.87⋅10^-01^ | 9.22⋅10^-05^ | *RFX4* |
|  | rs1828384 | 115575730 | C | 7.67⋅10^-03^ | 1.62⋅10^-06^ | 7.06⋅10^-05^ |  |
| 13 | rs9522923 | 90952373 | C | 1.63⋅10^-04^ | 5.41⋅10^-02^ | 8.01⋅10^-07^ |  |
|  | rs1408885 | 90952780 | A | 5.02⋅10^-04^ | 1.81⋅10^-01^ | 2.36⋅10^-06^ |  |
|  | rs7995023 | 91007492 | G | 1.30⋅10^-02^ | 2.42⋅10^-01^ | 6.74⋅10^-05^ |  |
|  | rs9515738 | 91031065 | G | 7.86⋅10^-05^ | 5.27⋅10^-02^ | 8.91⋅10^-06^ |  |
| 14 | rs17095160 | 97766226 | G | 9.06⋅10^-05^ | 1.59⋅10^-06^ | 4.39⋅10^-05^ |  |
| 15 | rs17532319 | 36243624 | T | 1.33⋅10^-04^ | 3.88⋅10^-01^ | 6.00⋅10^-05^ |  |
| 19 | rs11668201 | 59003632 | T | 4.21⋅10^-03^ | 9.15⋅10^-01^ | 2.35⋅10^-05^ |  |
|  | rs11669127 | 59003830 | T | 6.75⋅10^-03^ | 9.75⋅10^-01^ | 6.04⋅10^-05^ |  |
|  | rs3794963 | 59071051 | G | 3.27⋅10^-02^ | 8.61⋅10^-01^ | 6.37⋅10^-05^ |  |
| 20 | rs6076827 | 5374252 | G | 2.43⋅10^-02^ | 1.83⋅10^-06^ | 4.59⋅10^-05^ |  |

^a^Chromosome; ^b^Positions according to UCSC version hg19; ^c^Minor allele; ^d^Main effect of nuclear SNP; ^e^Main effect of mitochondrial SNP; ^f^Interaction term.
