# Supplementary material for: Male-specific association between MT-ND4 11719 A/G polymorphism and ulcerative colitis: a mitochondria-wide genetic association study
Source: BMC Gastroenterol. 2016 Oct 3;16:118. doi: 10.1186/s12876-016-0509-1 (PMC5048482; doi:10.1186/s12876-016-0509-1)
Supplement: Additional file 8: Table S5. — Results for nuclear SNPs with p value < 1·10−04 for interaction with rs2853495 in the subgroup of males, sorted by chromosomal positions. (DOC 78 kb) [file 12876_2016_509_MOESM8_ESM.doc]

**Table S5:** Results for nuclear SNPs with p value < 1∙10^-04^ for interaction with rs2853495 in the subgroup of males, sorted by chromosomal positions.

| Chr^a^ | Nuclear SNP | Position^b^ | A1^c^ | p value | | | Gene |
| --- | --- | --- | --- | --- | --- | --- | --- |
|  |  |  |  | nuclear^d^ | mito^e^ | interaction^f^ |  |
| 1 | rs6664866 | 11000721 | T | 2.36⋅10^-03^ | 3.88⋅10^-07^ | 4.61⋅10^-05^ |  |
|  | rs10800238 | 166456387 | T | 3.76⋅10^-03^ | 4.14⋅10^-07^ | 5.68⋅10^-05^ | *RP11-276E17.2* |
|  | rs7552571 | 166456476 | T | 3.12⋅10^-03^ | 5.31⋅10^-07^ | 4.92⋅10^-05^ | *RP11-276E17.2* |
|  | rs7552777 | 166456704 | T | 2.65⋅10^-03^ | 2.03⋅10^-07^ | 2.19⋅10^-05^ | *RP11-276E17.2* |
| 2 | rs12621507 | 105784318 | T | 1.55⋅10^-02^ | 5.58⋅10^-07^ | 5.77⋅10^-05^ |  |
|  | rs6747261 | 109510152 | C | 4.25⋅10^-05^ | 8.53⋅10^-06^ | 6.72⋅10^-05^ | *EDAR* |
|  | rs6746848 | 141471725 | G | 2.82⋅10^-03^ | 9.40⋅10^-07^ | 7.35⋅10^-05^ | *LRP1B* |
|  | rs11894546 | 150057912 | A | 1.04⋅10^-01^ | 1.67⋅10^-07^ | 1.92⋅10^-05^ | *LYPD6B* |
|  | rs415974 | 77637545 | T | 1.22⋅10^-02^ | 3.70⋅10^-01^ | 9.27⋅10^-05^ | *LRRTM4* |
| 3 | rs9820958 | 114353168 | A | 7.75⋅10^-03^ | 1.42⋅10^-05^ | 9.64⋅10^-05^ | *ZBTB20* |
|  | rs9841454 | 114362596 | C | 3.43⋅10^-02^ | 1.30⋅10^-05^ | 9.71⋅10^-05^ | *ZBTB20* |
|  | rs541855 | 127496076 | T | 2.21⋅10^-04^ | 8.84⋅10^-01^ | 5.07⋅10^-05^ | *MGLL* |
|  | rs586919 | 127496088 | C | 2.08⋅10^-04^ | 9.05⋅10^-01^ | 4.75⋅10^-05^ | *MGLL* |
|  | rs12638237 | 40063887 | G | 2.76⋅10^-02^ | 1.01⋅10^-04^ | 5.27⋅10^-05^ | *MYRIP* |
| 7 | rs3113304 | 68549929 | G | 5.88⋅10^-03^ | 1.24⋅10^-06^ | 9.31⋅10^-05^ |  |
| 10 | rs11016262 | 130266862 | T | 1.07⋅10^-01^ | 3.43⋅10^-01^ | 1.94⋅10^-05^ |  |
|  | rs4237441 | 14975171 | T | 2.67⋅10^-02^ | 2.12⋅10^-05^ | 5.06⋅10^-05^ | *DCLRE1C* |
|  | rs7091361 | 29855186 | G | 3.49⋅10^-03^ | 1.39⋅10^-01^ | 7.66⋅10^-05^ | *SVIL* |
|  | rs973286 | 29865078 | C | 2.92⋅10^-03^ | 1.61⋅10^-01^ | 8.92⋅10^-05^ | *SVIL* |
|  | rs10826663 | 29874104 | C | 3.00⋅10^-03^ | 2.82⋅10^-01^ | 9.06⋅10^-05^ | *SVIL* |
|  | rs11009199 | 33342328 | C | 2.92⋅10^-03^ | 4.32⋅10^-06^ | 6.67⋅10^-05^ | *RP11-462L8.1* |
|  | rs1106789 | 87553409 | G | 2.55⋅10^-03^ | 3.48⋅10^-07^ | 2.65⋅10^-06^ | *GRID1* |
|  | rs1880386 | 87611943 | T | 9.41⋅10^-03^ | 4.41⋅10^-06^ | 5.03⋅10^-05^ | *GRID1* |
| 11 | rs2703758 | 130368730 | C | 2.44⋅10^-03^ | 3.73⋅10^-06^ | 9.64⋅10^-05^ |  |
|  | rs11607974 | 17233864 | A | 1.39⋅10^-02^ | 6.32⋅10^-01^ | 1.76⋅10^-05^ | *NUCB2* |
|  | rs1425802 | 35158401 | C | 1.96⋅10^-02^ | 1.93⋅10^-06^ | 9.89⋅10^-05^ | *CD44* |
|  | rs7938811 | 35177552 | C | 7.01⋅10^-04^ | 2.87⋅10^-06^ | 3.76⋅10^-06^ | *CD44* |
|  | rs11037114 | 42889289 | T | 1.08⋅10^-01^ | 8.27⋅10^-07^ | 9.28⋅10^-05^ |  |
| 12 | rs11519533 | 17088421 | C | 1.92⋅10^-04^ | 3.38⋅10^-05^ | 5.11⋅10^-05^ |  |
|  | rs7311005 | 99204594 | G | 3.65⋅10^-02^ | 6.03⋅10^-01^ | 9.71⋅10^-05^ | *ANKS1B* |
| 13 | rs1218787 | 27961940 | T | 1.21⋅10^-02^ | 3.03⋅10^-06^ | 8.17⋅10^-05^ |  |
|  | rs9522923 | 90952373 | C | 7.15⋅10^-03^ | 3.36⋅10^-01^ | 8.69⋅10^-05^ |  |
|  | rs1408885 | 90952780 | A | 3.35⋅10^-03^ | 5.97⋅10^-01^ | 7.06⋅10^-05^ |  |
| 14 | rs3180753 | 95659747 | T | 8.60⋅10^-03^ | 9.99⋅10^-07^ | 4.50⋅10^-05^ | *CLMN* |
| 15 | rs10444830 | 53797943 | C | 4.59⋅10^-02^ | 8.63⋅10^-01^ | 2.25⋅10^-05^ |  |
|  | rs12440270 | 53804400 | C | 6.45⋅10^-02^ | 9.12⋅10^-01^ | 4.04⋅10^-05^ | *WDR72* |
|  | rs12440842 | 53824229 | T | 3.80⋅10^-02^ | 9.31⋅10^-01^ | 7.04⋅10^-05^ | *WDR72* |
| 16 | rs2369690 | 22920852 | A | 4.48⋅10^-02^ | 1.70⋅10^-05^ | 2.90⋅10^-05^ | *HS3ST2* |
|  | rs9935276 | 24242392 | A | 4.71⋅10^-03^ | 6.16⋅10^-01^ | 3.86⋅10^-06^ |  |
| 17 | rs7225156 | 1895906 | G | 1.25⋅10^-03^ | 5.11⋅10^-02^ | 4.50⋅10^-06^ | *RTN4RL1* |
| 21 | rs2839575 | 44126116 | A | 3.74⋅10^-01^ | 3.80⋅10^-07^ | 4.70⋅10^-05^ | *PDE9A* |

^a^Chromosome; ^b^Positions according to UCSC version hg19; ^c^Minor allele; ^d^Main effect of nuclear SNP; ^e^Main effect of mitochondrial SNP; ^f^Interaction term.
